# Emerging Electroencephalographic Biomarkers to Improve Preclinical to Clinical Translation in Alzheimer’s Disease

**DOI:** 10.3389/fnagi.2022.805063

**Published:** 2022-02-17

**Authors:** Zackary A. Cope, Takeshi Murai, Stacey J. Sukoff Rizzo

**Affiliations:** Aging Institute, School of Medicine, University of Pittsburgh, Pittsburgh, PA, United States

**Keywords:** EEG, Alzheimer’s disease, animal models, mice, non-human primate, biomarkers

## Abstract

Continually emerging data indicate that sub-clinical, non-convulsive epileptiform activity is not only prevalent in Alzheimer’s disease (AD) but is detectable early in the course of the disease and predicts cognitive decline in both humans and animal models. Epileptiform activity and other electroencephalographic (EEG) measures may hold powerful, untapped potential to improve the translational validity of AD-related biomarkers in model animals ranging from mice, to rats, and non-human primates. In this review, we will focus on studies of epileptiform activity, EEG slowing, and theta-gamma coupling in preclinical models, with particular focus on its role in cognitive decline and relevance to AD. Here, each biomarker is described in the context of the contemporary literature and recent findings in AD relevant animal models are discussed.

## Introduction

Alzheimer’s disease (AD) is the foremost cause of dementia (NIA Alzheimer’s Disease Fact Sheet, 2020) and the fifth leading cause of death worldwide. Over 6 million people 65 or older currently live with AD (WHO, 2020). As global life expectancy continues to increase this number of patients is expected to double every 20 years and reach 131.5 million by 2050 (Alzheimers.net, 2020). The global cost of dementia is expected to exceed $2 trillion United States by 2030, topping the gross domestic product of all but 17 countries and the total market values of Apple, Google, and Exxon. At this rate, AD will place increasingly severe burdens on global healthcare markets over the next 30 years. People living with dementia also require caregiver assistance, especially in the later stages of the disease. Frequently, these caregivers are close relatives (World Alzheimer Report, 2015). Global cost estimates will never fully capture the physical and emotional toll imposed on these individuals who love and care for someone suffering from AD.

It is now over 115 years since the first clinical report describing AD in 1906 (Alzheimer, 1906), but the first purported “disease-modifying” treatment for AD, Aduhelm was only recently approved in June 2021 (Yang and Sun, 2021). Time will tell whether this new treatment can make an impact. To date, the search for a cure has largely focused on clearance and prevention of histopathological aggregates such as amyloid plaques (Haass and Selkoe, 2007; Mucke and Selkoe, 2012; Selkoe and Hardy, 2016) and neurofibrillary inclusions. More recently, investigation has broadened to strategies for minimizing chronic inflammation, mediating neurovascular risk-factors (NIA, What Happens to the Brain in Alzheimer’s Disease, 2020), vaccination (Novak et al., 2017; Kabir et al., 2020), and development of exogenous antibodies (Sevigny et al., 2016; Uhlmann et al., 2020). These interventions continue to fall short of meeting the most important endpoint of AD, which is improvement or prevention of cognitive decline in AD. In conjunction with symptom modifying drugs such as acetylcholinesterase inhibitors or low-affinity NMDA blockers such as memantine (Galimberti and Scarpini, 2011), prevention and early intervention remain the most viable current treatment strategies.

Early detection and treatment at the very earliest stages of amyloid seeding may hold critical potential for the treatment of AD (Uhlmann et al., 2020). Owing to its widespread accessibility, data-rich non-invasive methods, and low cost relative to expensive diagnostic neuroimaging, electroencephalography (EEG) is a promising approach for detection and development of early biomarkers related to neurological dysfunction resulting from AD. In particular, seizure and sub-clinical epileptiform activity related to AD is readily identifiable with EEG (Horváth et al., 2016; Palop and Mucke, 2016; Lam et al., 2017; Horvath, 2018; Smailovic et al., 2018). Recent reports indicate that sub-clinical, non-convulsive epileptiform activity is not only prevalent in AD (Palop and Mucke, 2016; Vossel et al., 2016, 2017; Horváth et al., 2017; Lam et al., 2017), but is detectable early in the course of the disease (Vossel et al., 2013), and predicts cognitive decline (Vossel et al., 2016). Given that preclinical translational studies focusing on behavioral outcomes for cognition have repeatedly failed to translate to cognitive benefits in humans, there is an urgent need to develop improved translational approaches. To this end, advances in measuring epileptiform activity and other EEG biomarkers may hold powerful, untapped potential to improve the translational validity of AD-related biomarkers in model animals and enable improved preclinical to clinical translation for understanding cognitive deficits and potential therapeutic interventions for AD.

## Mechanisms Underlying Abnormal Epileptiform Activity

Epileptiform activity is a broad term to describe sporadic discharges or trains of spiking activity resulting from highly synchronous oscillatory activity in the brain. The activity captured in a surface EEG is largely generated from reciprocal excitatory-inhibitory connections throughout thalamocortical circuits and is not necessarily equated to classical full body seizure type events; although severe events of this nature may result in such. In short, hyperactivity in layer VI pyramidal cells triggers an inhibitory response from GABAergic neurons within the thalamic reticular nucleus. During this inhibitory response, low-threshold calcium channels in thalamocortical neurons recover from initial inactivation. Consequently, as thalamocortical neurons recover from inhibitory post-synaptic currents generated by the thalamic reticular nucleus, this available pool of calcium channels trigger another burst of excitatory activity. This cycle produces reverberating oscillatory activity detectable as spike trains in EEG (Blumenfeld, 2005). Perturbations in this excitatory-inhibitory (E-I) balance, particularly within cortico-hippocampal circuitry could arise from innumerable gene mutations or physical and environmental insults in the polygenetic or degenerative patterns in late onset AD (LOAD). In LOAD, constellations of genetic mutations converge to produce synapse deterioration and loss, amyloidosis, neurotoxicity, and neuroinflammation that can all contribute to shifting the intrinsic E-I balance.

Current theories hypothesize that progressive age-related decline of synaptic function in the earliest stages of AD alters firing properties of neurons within broad networks mediating genome, immune, and metabolic homeostasis, among other functions. Dysregulation within these networks feeds forward resulting in loss of function and cognitive decline as in AD (Frere and Slutsky, 2018). This understanding of homeostatic dysfunction resulting from age-related degradation of synaptic function across broad regulatory networks captures the diversity of genetic and environmental factors at play in sporadic AD, while also speaking to mechanisms of autosomal dominant familial AD (FAD) mutations.

## Incidence and Prevalence

Despite broad acceptance of its occurrence, accurate coincidence of abnormal epileptiform activity in AD has proven difficult to estimate. Given that these neural perturbations are often subclinical and not presented with any overt motoric type of seizure events, awareness of seizure activity may be limited. Indeed, previous reviews have reported incidence ranging from 0.5 to 64% (Horváth et al., 2016; Palop and Mucke, 2016). However, two separate studies recently reported incidence rates of 13.4 and 14.0% in LOAD patients (Beagle et al., 2017; Lyou et al., 2018). This broad range may result from the diversity of seizure types assessed in each study. Seizure recurrence is frequent, increases with disease duration, and is associated with poorer cognitive performance (Vöglein et al., 2020). While convulsive, tonic-clonic seizures which are easily detected by overt motor phenomena has largely comprised a bulk of the literature related to reports of seizures in AD to date (Horváth et al., 2016). By comparison, a recent study reported 42.4% of patients meeting the National Institute of Aging – Alzheimer’s Association criteria for probable AD (McKhann et al., 2011) demonstrated epileptiform activity. Interestingly, all patients in this study also showed evidence of cortical atrophy assessed using voxel-based morphometry with amyloidosis confirmed in 61% of these patients by PET/MR. Of those not exhibiting positive amyloid PET, several tested positive for CSF biomarkers Aβ42, t-Tau, p-Tau, or Aβ42-Tau index. Interestingly, at the time of initial EEG assessment patients presenting with epileptiform activity did not differ in global cognitive ability assessed by the Mini-Mental State Examination. Longitudinal monitoring of these patients over the course of 1–5 years revealed a more precipitous decline in global cognition and executive function (Vossel et al., 2016). These data therefore implicate epileptiform activity as an early indicator of AD pathology and future cognitive decline. Separately, another study comparing MCI and AD patients with epilepsy to AD patients with subclinical epileptiform activity reported that the latter group began experiencing cognitive decline 5.5–6 years sooner (Vossel et al., 2013). Implanted electrodes in two patients targeting the medial temporal lobe detected hyperexcitability in the region of the hippocampus that was not readily detectable on scalp electrodes. This activity was especially prominent during sleep (Lam et al., 2017). Continuous EEG monitoring over a 24 h period in 5 AD patients with epilepsy, revealed 82% of epileptiform discharges occurred during sleep, with 55% occurring during non-rapid eye movement (NREM) sleep (Horváth et al., 2017). Interference with memory consolidation that takes place during this period likely contributes to continued cognitive decline in AD patients. These finding are not without challenges, however, as analysis of seizure activity using more strictly defined parameters has failed to detect differences in subclinical seizure incidence between cases and controls (Brunetti et al., 2020). Further study should seek to define specific criteria for subclinical epileptiform activity that drives differences between cases and controls. Taken together, these clinical data indicate that epileptiform activity may indeed serve as an early biomarker for cognitive decline and early pathological events in AD.

## Translational Electroencephalographic Biomarkers for Preclinical Investigation in Animal Models

Recent literature highlights the utility of EEG studies in animal models as demonstrating translational potential for AD (Born, 2015; Horváth et al., 2016; Palop and Mucke, 2016; Vossel et al., 2017). More specifically, four distinct phenotypes have emerged that include (1) ectopic cortical spiking activity, especially during NREM sleep, absent overt physical symptoms of acute seizure, (2) hippocampal slowing evidenced by leftward shifts in the power distribution of EEG oscillatory activity at baseline, (3) hyperexcitability of hippocampal neurons, particularly in response to cortical inputs, and (4) impaired theta-gamma coupling. In patient populations, this activity is not only highly predictive of disease course (Vöglein et al., 2020), but may precede the earliest cognitive symptoms. Here we review these outcome measures, identifying reports from relevant animal models expressing these phenotypes and their translational utility for AD. While previous publications have discussed epileptiform and other abnormalities in EEG activity in AD animal models generally focusing on FAD alleles which can be comprehensively reviewed elsewhere (Born, 2015; Horváth et al., 2016; Palop and Mucke, 2016; Vossel et al., 2017; Kitchigina, 2018), the present review provides a contemporary update that highlights subsequent and more recent findings in these and newer mouse models with a focus on distinct phenotypes of EEG outcome measures.

In animal models, epileptiform activity is used broadly to refer to ectopic discharges that may present with (interictal/seizure) or without (subclinical) motor features. Applications of EEG in animal models generally consists of the identification of spike wave discharges (SWD) or single spikes predominantly during NREM. SWDs are typically characterized by surface-negative spikes alternating with 7–10 Hz surface positive waves (Jin et al., 2021). Single spikes, as their name would imply are sharp deflections in the EEG trace that do not cluster with other spikes as in an SWD. This type of spike has no clear definition, owing to the complexity and heterogeneity of this type of activity. These spikes are generally quantified as events crossing a predefined threshold relative to baseline activity of the EEG trace. These criteria vary widely, but general guidelines have recently been developed for mice which will greatly facilitate improved rigor and reproducibility going forward (Jin et al., 2021).

Electroencephalographic slowing is a key biomarker in AD and AD related dementias (ADRD) (Jeong, 2004; Horvath, 2018). In the EEG trace, EEG slowing generally manifests as a reduction in the distribution of alpha (8–12 Hz) and beta (12–30 Hz) waves and a commensurate increase in the distribution of delta (0.5–4 Hz) and theta (4–8 Hz) waves (Brenner et al., 1986; Jeong, 2004). Patients with MCI exhibit increases in theta activity and a decrease in beta activity. Severe dementia, on the other hand, is related to decreases in alpha and an increase in delta frequency activity (Coben et al., 1985). Lower overall mean frequency is also well correlated to dementia severity (Brenner et al., 1986). EEG slowing is likely attributable to degeneration of acetylcholine neurons during the course of AD (Bartus et al., 1982). This is supported by multiple clinical and preclinical studies using the muscarinic antagonist scopolamine (Ebert and Kirch, 1998). In rats administration of scopolamine increases the power in the delta and theta range (Buzsáki et al., 1988). In healthy human subjects scopolamine administration increased delta and theta activity while decreasing alpha and beta power (Ebert et al., 2001). Further, scopolamine is well known for inducing memory impairment in healthy subjects, akin to impairment measured in MCI (Ebert and Kirch, 1998).

Hyperexcitability of hippocampal neurons has been used in animal models as a surrogate to hippocampal hyperactivation observed in AD patients during fMRI (Zott et al., 2018). In an fMRI, memory tasks increase hippocampal activation with hyperactivity reported in AD patients reported prior to and throughout initial impairments in cognitive performance and mild memory loss (Dickerson et al., 2005; Huijbers et al., 2015). Later in the progression of AD as cognitive impairment becomes more severe, hippocampal activity is diminished (O’Brien et al., 2010). This pattern may suggest an initial overcompensation by the hippocampus followed by a loss of activation later in the course of AD progression (Sperling et al., 2010).

Theta-gamma coupling (TGC) is another electrophysiological biomarker with emerging relevance to AD (Goodman et al., 2018; Kitchigina, 2018). TGC refers to the modulation of gamma oscillations (30–100 Hz) by slower theta oscillations (4–7 Hz) This relationship was first characterized in the study of place cells in the hippocampus. Specifically, place cells were shown to fire at different theta phases according to a specific temporal pattern coinciding with sequential spatial position on a walking track. Therefore, theta activity encodes information along a temporal sequence, termed temporal sweeps. Gamma oscillations “nested” within each temporal sweep are thought to represent units of events in order. In other words, theta represents the time interval during which items of information encoded by gamma are held in memory. The extent which gamma varies as a function of theta is quantified as TGC (Lisman and Buzsáki, 2008; Lisman and Jensen, 2013). Breakdowns in this encoding would lead to degradation of representations of events in order. Indeed, this type of cross-frequency coupling is important for both long-term (Tort et al., 2009) and working memory (Lisman, 2010). Patients with AD show significantly lower TGC compared to healthy controls, which is associated with impaired performance on an N-back task (Goodman et al., 2018).

## Epileptiform Activity in Alzheimer’s Disease-Relevant Mouse Models

Much of the preclinical AD research to date has utilized mouse models expressing FAD mutations, specifically amyloid precursor protein (APP) and presenilin 1 (PS1). Despite the causal mechanisms of these mutations in early-onset AD, FAD only comprises between 1 and 5.5% of all AD cases (Sassi et al., 2014; Zhu et al., 2015). Moreover, severe neuropathology and behavioral phenotypes are detectable in young FAD mice, a result of transgenic overexpression that is well beyond physiologically relevant changes (Oddo et al., 2003; Richard et al., 2015) limiting translational value for the majority of AD cases who do not experience rapid decline in early adulthood. New approaches seek to incorporate LOAD risk-factor genes in tandem with FAD mutations, other risk alleles, and environmental variables to begin understand the complex age-dependent etiology of AD that has remained elusive to date (Onos et al., 2016; Castillo et al., 2017; Pandey et al., 2019; Preuss et al., 2019; Oblak et al., 2020). Understanding how these newly emerging risk-factors play a role in the induction of epileptiform activity and network dysfunction could provide key insights to earlier sub-clinical manifestations of LOAD.

Amyloid precursor protein is a transmembrane protein with yet unknown endogenous functions, though it has been implicated in a broad array of roles such as synapse formation (Priller et al., 2006), plasticity (Montagna et al., 2017), and metallic ion chelation (Cuajungco et al., 2005). Proteolysis of APP is accomplished through two pathways. Initial cleavage is executed by α- or β-secretase. Cleavage by β-secretase, also known as Beta APP Cleaving Enzyme 1 (BACE1) produces β-amyloid (Aβ) fragments after final cleavage by γ-secretase. These fragments are the primary component of amyloid plaques in AD (Nunan and Small, 2000). Many FAD mutations of APP have been characterized, which has led to the generation of equally diverse APP mouse models.

One of the earliest APP mouse models is the Tg2576, overexpresses a mutant form of APP with the Swedish (APP KN670/671NL) mutation resulting in significant and early onset amyloid pathology. This is a double mutation directly adjacent to the β-cleavage site of APP, which facilitates β-secretase cleavage. Owing to its name, the Swedish mutation was first characterized in two Swedish families with a high degree of genealogical overlap. Affected members of these families experienced progressive memory loss consistent with AD (Mullan et al., 1992). Late in the disease course, myoclonic jerks and seizures are frequent in these families (Axelman et al., 1994). Aβ plaque aggregation is present in aged Tg2576 mice (>11 months), though diminished LTP (Jacobsen et al., 2006) and synapse loss (Lanz et al., 2003) has been described in 4–5 months old mice before plaques are detected (Table 1). Cognitive impairments in spatial learning and fear conditioning were initially thought to be progressive, only seen in 12 months or older mice (Hsiao et al., 1996). Though subsequent reports have describe behavioral deficits as early as 6 months (Jacobsen et al., 2006). Recent publications have characterized large, synchronous spikes consistent with interictal spikes seen in epilepsy. These spikes are present in 5 weeks old mice, long before any of the pathology discussed. These spikes progressed into adulthood (7 months) and were predominantly detected during REM sleep (Kam et al., 2016). Though it is unclear if the exogenous transgene hamster prion protein promotor itself be disrupting neuronal signaling and contributing to the epileptiform activity in these animals. It should be noted that Tg2576 mice are maintained on a hybrid C57BL6/SJL background. Lower seizure thresholds have been reported for the SJL mouse (Golden et al., 2001) and depending on C57 or SJL substrain used, this may contribute to SWD phenotype as well (Letts et al., 2014) which underscores the need for specific documentation of background strain given the heterogeneity of seizure phenotypes across AD transgenic lines and mouse strains and substrains (Schauwecker, 2011; Letts et al., 2014; Copping et al., 2019).

**TABLE 1:** Summary of rodent models, Alzheimer’s disease relevant pathology and reported behavioral and EEG phenotypes.

Model system	Amyloidosis	Electrophysiological biomarkers	Other abnormalities	Behavioral phenotypes	Therapeutic assessment
Tg2576	Present >11 months of age.	Interictal spikes as early as 5 weeks of age progressing into adults.	Diminished LTP, synapse loss (4–5 months), overexpression of NaV channels.	Spatial learning and fear conditioning deficits at 6 months of age.	Not reported.
J20	Present by 6 months.	Non-convulsive spiking activity detected as early as 1 month of age.	Neuron and synapse loss, diminished LTP/LTD present by 3 months.	Spatial reference memory impairment by 4 months old, increased open arm time in EPM.	Levetiracetam suppressed seizure, blocked increased neurogenesis, rescued spatial memory deficits. No response to BACE-1 inhibitor NB-360.
App*^NL–G–F^*	Aggressive, present by 3 months of age.	Non-convulsive spiking activity present in spike-waves.	Gliosis and synapse loss in cortex and hippocampus from 2 months.	Spatial reference memory impairment by 4 months, increased open arm time in EPM.	Not reported.
APP*^NL/F^*	Present by 6 months, lower relative to J20.	Not detected.	Gliosis present by 6 months, synapse loss from 9 months.	Spatial memory deficits in y-maze, but not MWM at 18 months old.	Not reported.
APP/PS1	Detectable at 6 weeks old in cortex, 3–5 months in hippocampus.	Diverse, including interictal spikes, polywaves, and sharp waves.	Deficits in CA1 LTP from 7 months. Limited neuron loss in hippocampus at 17 months old.	Deficits in MWM spatial memory.	Epileptiform activity precipitated by physostigmine. Pentylenetetrazole increased evoked seizure susceptibility.
APP/PS1ΔE9	Progressive beginning from 6–12 months.	Multiple spike phenotypes, notably giant spikes.	Impaired LTP and gliosis at 3 months old, synapse loss at 4 months.	Impairments in MWM present at 12–13 months, but not 7 months.	Giant spikes reduced by levetiracetam and ethosuximide. Seizures increased by fluoxetine and paroxetine.
3xTg-AD	Detectable at 6 months.	Hippocampal hyperexcitability associated with increased theta power. Hypersynchronous activity between hippocampus and cortical regions.	Tau aggregation and diminished LTP at 6 months, gliosis present at 7 months.	Impaired MWM and contextual fear conditioning from 4 to 6 months.	Hippocampal theta increase is normalized with K-channel antagonist 4-aminopyradine. Hyperexcitability attenuated with anti-human APP antibody or mGluR5 antagonist.
5xFAD	Present at 2 months of age.	Ictal discharges occurring during NREM sleep. Increased hippocampal theta.	Gliosis present at 2 months old. Loss of layer V pyramidal neurons and synapses present by 6 months old.	General hyperactivity, motor and balance impairments at 9 months, increased social interaction and deficits in spontaneous alternation and MWM progressing from 4 months old.	Not reported.
APOE4 TR	Measured at 9 months, interacting with LDL receptor-related protein.	Motoric jerks associated with frequent cortical spiking, increased hippocampal theta.	Diminished LTP and longer duration field potentials in hippocampal slices, decreased dendritic spine density.	Spatial learning and memory deficits in Barnes Maze and MWM present at 3 months persisting to 18 months.	Not reported.
ADAM10	Not reported.	KO of ADAM10 was related to increases in hippocampal alpha and theta power.	Increased neuroinflammation with overexpression, increased gliosis and loss of spine morphology in KO.	Behavioral deficits characterized in APP/PS1 mice are modulated by overexpression or KO of ADAM 10.	Not reported.
α-synuclein	Not reported *in vivo.*	Overexpression of human gene related to myoclonic jerks, ictal spikes, and leftward shift in EEG power.	Reduced calbindin expression in hippocampus, increased neuropeptide Y.	Not reported.	Not reported.
F344-AD Rat	Present from 6 months of age.	Evoked theta oscillations present in hippocampus at 6 months old lost by 12 months old.	Gliosis present at 6 months old. Neuron loss in cortex and hippocampus by 16 months old.	Reversal learning deficits characterized using MWM.	Evoked theta activity restored at 12 months by treatment with donepezil.

A recent paper by Johnson et al. (2020) directly compared epileptiform activity across three APP mouse models. hAPP-I5 (I5) mice carry a transgene overexpressing human neuronal APP (hAPP). This mouse serves as a comparison strain for FAD-mutant hAPP mice (J20), which overexpress APP with Swedish (KN670/671NL) and Indiana (V717F) mutations (Mucke et al., 2000). The third strain, App*^NL–G–F^*, does not overexpress APP, but instead express fully humanized Aβ fragments from cleavage of Swedish (KM670/671NL), Arctic (E693G), and Beyreuther/Iberian (I716F) mutant APP (Saito et al., 2014). As expected, I5 and J20, but not APP*^NL–G–F^*, overexpress APP in cortex and hippocampus at 6–8 months of age. Though, compared to I5 and J20 strains, APP*^NL–G–F^* express more Aβ overall with a higher ratio of Aβ_1–42_ in hippocampus and cortex. EEG recordings in all three of these mouse models at 8–12 months of age revealed non-convulsive epileptiform activity compared to their WT littermates. J20 mice expressed the most spikes over the entire 12 h recording, while the APP*^NL–G–F^* mice experienced the highest number of spike wave discharges. Behaviorally, all three strains displayed deficits in active place avoidance. However, given that J20 and APP*^NL–G–F^* mice also spent more time in the open arms of an elevated plus maze (EPM) indicative of an anxiolytic-like phenotype, it may be difficult to distinguish between spatial memory deficits in an avoidance paradigm or an overall loss of inhibition/aversion to risk in these mice. Subsequent experiments showed that J20 mice do exhibit spatial memory deficits in the Morris Water Maze (MWM) (Johnson et al., 2020), although the MWM phenotype in APP*^NL–G–F^* mice has been inconsistent (Mehla et al., 2019; Saifullah et al., 2020). However, deficits in location discrimination, in the absence of MWM deficits have been detailed (Saifullah et al., 2020). Spatial memory deficits (Cheng et al., 2007; Wright et al., 2013), neuron loss (Wright et al., 2013), and LTP deficits (Saganich et al., 2006) have been reported in J20 mice as early as 4 months of age. Thus, it remains to be determined if epileptiform activity in J20 mice is a cause or effect of the early pathology seen in this strain, or if promoter driven transgene expression may be playing a role on its own. Notably, treatment with the BACE1 inhibitor NB-360 did not modify any of the behavioral or synaptic abnormalities measured in the J20 mice despite lowering levels of Aβ (Johnson et al., 2020). This particular result is notable as BACE1 inhibitors have failed to translate to improved cognition in the clinic, despite AB lowering (Mullard, 2018). Overall, these results indicate that APP overexpression is not necessary for inducing non-convulsive epileptiform activity or behavioral abnormalities induced by FAD mutations (Johnson et al., 2020). In a separate study comparing J20 to APP*^NL/F^* mice that carry the Swedish and Iberian APP mutations (Saito et al., 2014; Brown et al., 2018), epileptiform activity in the form of inter-ictal spikes was identified in J20 mice, especially during REM sleep. These events were not detected in APP*^NL/F^* mice. Interestingly, APP*^NL/F^* mice express more physiologically relevant levels of Aβ compared to overexpression in J20 and APP*^NL–G–F^* strains, which may diminish their seizure susceptibility although it remains unclear any direct correlations of Aβ levels with seizure susceptibility in humans or mouse models. Importantly, these findings during REM sleep in APP models contradict the predominance of epileptiform activity in NREM sleep in humans, suggesting key differences in seizure pathology across species not yet understood (Kam et al., 2016; Brown et al., 2018). Additional recent studies have begun to shed light on seizure-related pathogenesis in J20 mice. Increased spike wave discharges and increased latency of field responses in reticular thalamus following stimulation in deep cortical layers have been characterized from 4 to 6 months (Hazra et al., 2016). Reports that amyloid deposition leads to large increases of A_2*a*_ expression on astrocytes suggests that in these J20 mice, astrocytic adenosine A_2*a*_ receptors may play a role in AD like pathology. The A_2*a*_ blocker istradefylline remediated MWM deficits following 1–3 weeks treatment administered in drinking water (Orr et al., 2018). Activation of A_2*a*_ is proconvulsant and decreases seizure thresholds in kindling procedures (Tescarollo et al., 2020). These preclinical data open interesting avenues for studying the relationship of A_2*a*_ receptors related to spontaneous seizure incidence in AD.

Over 70 mutations of the presenilin (PSEN) gene have been characterized (Jankowsky et al., 2004), accounting for roughly 90% of all FAD cases (Watanabe and Shen, 2017). The PS genes, PS1 and PS2, encode the catalytic subunit of γ-secretase, the final enzyme involved in APP proteolysis. When APP is initially cleaved by β-secretase, final cleavage by γ-secretase produces amyloid fragments typically between 38 and 42 residues in length, with Aβ40 comprising the bulk of amyloid fragments produced by APP proteolysis through the β-secretase pathway. Mutations in PSEN can result in greater production of long amyloid fragments, particularly Aβ42 that has a higher liability for deposition and, therefore produces more amyloid plaques compared to shorter fragments. This greater plaque burden is key in the pathogenesis of PSEN related FAD (Chow et al., 2010).

The APP/PS1 mouse combines a transgene carrying both hAPP and PSEN mutations, though the specific mutations may vary per each strain. Mice expressing APP Swedish and PSEN L166P mutations are most common. These mice express the APP transgene at threefold higher levels compared to murine APP and human Aβ42 is expressed in higher concentrations over Aβ40. Amyloid deposition is detectable in 6 weeks old mice, beginning in the neocortex. Amyloid deposits in the hippocampus, striatum, thalamus and brainstem appear in young adult mice 3–5 months old, though tau inclusions have not been identified in this strain (Radde et al., 2006; Maia et al., 2013). Deficits in spatial reversal learning, MWM spatial memory, and CA1 LTP have been observed as early as 7 months of age (Radde et al., 2006; Serneels et al., 2009; Gengler et al., 2010). Neuron loss is minimal in APP/PS1 mice and is generally constrained to the dentate gyrus as measured in 17-month old mice (Rupp et al., 2011). Spontaneous epileptiform discharges including interictal spikes, polywaves, and sharp waves have been described in 4–9 months old APP/PS1 mice, which were precipitated by the acetylcholinesterase inhibitor physostigmine (Reyes-Marin and Nuñez, 2017). These mice also have a greater susceptibility to seizures evoked using the pro-convulsant pentylenetetrazole, which was strongly correlated to the number of Aβ plaques measured in the cortex (Reyes-Marin and Nuñez, 2017).

Another study comparing mice carrying a deletion of PSEN1 exon 9 (APP/PS1ΔE9) also reported a seizure phenotype exhibiting multiple forms of spontaneous epileptiform events. This is particularly interesting because indeed patients with the PSEN1 exon 9 mutation also demonstrate seizure activity (Cortini et al., 2018). Utilizing electrodes implanted in cortical and subcortical structures, both strain dependency and structural dynamics of 8 specific epileptiform events could be identified. Three types of cortical spikes were identified. Cortical spikes were recorded when a discharge was detected across only cortical electrodes. These cortical spikes could co-occur with muscle twitches in the neck electromyogram and/or hippocampal discharges. Cortical spiking activity was also detected in sleep-spindles as well as higher frequency, low amplitude spindles (fast spindles). Spike wave discharges and cortico-hippocampal spikes with after-hyperpolarization were also characterized. The eighth type of discharge, termed a “giant spike” by the authors, was characterized using four criteria: (1) detection across all recording channels, (2) presence of spike complexes across multiple channels, (3) voltage changes ≥10 mV in hippocampal channel, and (4) a long after-hyperpolarization ≥10 ms. These giant spikes only occurred during sleep. APP/PS1ΔE9 mice experienced more cortical spikes either with or without hippocampal spikes, fast-spindles, cortico-hippocampal spikes, and giant spikes. In each case, these events were suppressed by treatment with the anticonvulsant levetiracetam or ethosuximide, a drug developed to suppress absence seizures. Notably, it was the presence of giant spikes that predicted seizure incidence in APP/PS1ΔE9 mice, demonstrating high utility of pairing cortical EEG with depth recording in hippocampus (Gureviciene et al., 2019). This is a particularly noteworthy translational phenotype given recent advances of foramen ovale recordings in distinguishing silent hippocampal seizures in AD (Lam et al., 2017).

Interestingly, a similar strain carrying the APP Arctic mutation (E693G) with the dE9 deletion have a comparatively mild seizure phenotype compared to the Swedish (APP KN670/671NL) mutation of APP. This mutation of APP also does not produce amyloidosis (Saito et al., 2014). This would suggest that seizure related activity is closely linked to the overproduction of amyloid, as in Swedish (APP KN670/671NL), whereas the APP Arctic mutation (E693G) may be more closely tied in with the pro-inflammatory phenotypes in AD.

Two separate papers have recently described a startling phenomenon in APP/PS1ΔE9 mice regarding the use of selective serotonin reuptake inhibitors (SSRIs) and the induction of AD-related seizure activity. Treatment with fluoxetine (Sierksma et al., 2016) and paroxetine (Severino et al., 2018) both led to increased age-related seizure incidence and premature death in these mice compared to their untreated controls. This effect is believed to arise from the anticholinergic properties of these drugs. Modern SSRIs seek to minimize their anticholinergic properties, which are associated with the most poorly tolerated side effects of modern anti-depressants, such as sedation, dry mouth, constipation, loss of libido and sexual dysfunction (Ferguson, 2001). Exposure to antidepressant, urological, or antiparkinsonian drugs with anticholinergic effects has been shown to induce anticholinergic cognitive burden that was related to increased incidence of dementia. This is particularly concerning considering the renewed interest in the anti-cholinergic mechanisms of rapid-acting antidepressants such as scopolamine (Drevets et al., 2013) and the increasing rates of antidepressant use over the last 20 years (Pratt et al., 2017). These data in mouse models provide further support for the use of EEG to identify potential adverse properties of therapeutic interventions.

The 5xFAD mouse has rapidly gained widespread use in the study of AD. These mice express 5 human transgenes linked to familial AD: three APP mutations [Swedish (K670N/M671L), Florida (I716V) and London (V717I) and two PSEN1 mutations (M146L and L286V)]. AD-relevant pathology in these mice progresses aggressively. Amyloid plaques and gliosis have been characterized in as early as 2 months old mice (Oakley et al., 2006). At 6 months, these mice are already experiencing neuron loss around areas with the highest plaque burden (Devi and Ohno, 2010). Despite prominent neuropathology, behavioral abnormalities have been inconsistent (Oblak et al., 2021). The most robust phenotypes to emerge are motor (hyperactivity) and balance impairments at 9 months (O’Leary et al., 2020), aggressive social interaction (Flanigan et al., 2014), spatial working memory deficits in spontaneous alternation at 4–5 months (Oakley et al., 2006; Devi and Ohno, 2010), and MWM deficits progressively declining from 6 months (Flanigan et al., 2014; Xiao et al., 2015; O’Leary et al., 2020). Though it is unclear how much of these motor and cognitive dysfunctions may be attributable to a general hyperactive behavioral phenotype (Flanigan et al., 2014; Lee et al., 2019; O’Leary et al., 2020). Neurophysiology impairments include degeneration of CA1 LTP (Kimura and Ohno, 2009) and loss of synaptic transmission in layer V cortex beginning at 6 months (Crouzin et al., 2013).

Siwek et al. (2015) characterized AD-relevant epileptiform activity, concluding that 18 months old 5XFAD mice exhibit ictal discharges in surface cortical EEG. However, EMG recordings were not included in this publication, making it difficult to distinguish these discharges from epochs of normal NREM sleep using the identification parameters reported (Siwek et al., 2015). As discussed, seizure activity in NREM is in fact typical in patients with AD (Horváth et al., 2016, 2017; Horvath, 2018). So while it is likely ictal discharges would occur in NREM, further characterization of sleep staging would help distinguish these events from normal sleep. Further, these mice were on a C57BL6/SJL background, which, as has been discussed previously, may alter seizure susceptibility (Golden et al., 2001; Letts et al., 2014). More recently, a rigorous comprehensive phenotypic characterization of 5XFAD on a congenic C57BL/6J background strain revealed a lack of robust EEG phenotypes in 5XFAD mice relevant to age and sex matched controls despite significant amyloid deposition in this model (Oblak et al., 2021). Single spikes have been identified as early as 4 months of age in 5xFAD mice. Interestingly in this paper it was found that spiking activity was exacerbated in an aquaporin KO 5xFAD mouse (Abe et al., 2020).

The ε4 allele of apolipoprotein E protein (APOE) is the strongest known risk factor for the development of non-familial LOAD. Three allelic variants of APOE are common in humans: ε2, ε3, and ε4. APOE is synthesized in the liver and the brain and mainly functions in the transport and homeostasis of cholesterol and other lipids throughout the body. With particular relevance to AD, APOE binds Aβ in the extracellular space and plays a role in clearing Aβ from the CNS through the blood–brain barrier (BBB). Of the three APOE isoforms, ApoE4 is poorest at binding Aβ and clearing it across the BBB; while APOE2 is the most effective. APOE2 is also known to be protective against AD. Thus, risk for LOAD is dose dependent on the APOE4 allele (APOE4/APOE4 > APOE3/APOE4 > APOE2/APOE4) (Kim et al., 2009; Holtzman et al., 2012; Safieh et al., 2019). APOE4 carriers have a higher risk for temporal lobe epilepsy (Najm et al., 2019), though it’s links to seizure in AD are still unclear.

An age-dependent seizure phenotype has been described in aged mice with a targeted replacement of the endogenous murine APOE allele with the humanized APOE4 allele (APOE4 TR). Female APOE4 TR mice were the first to develop a seizure phenotype of motoric jerks without arrest or clonus at six months of age. This same phenotype was present in males, albeit emerging later at 9 months. Cortical EEG reveled frequent spiking in the cortex of the APOE4 TR mice (Rodriguez et al., 2013).

Triggering receptor expressed on myeloid cells 2 (TREM2) is a frequent mutation related to AD. TREM2 is a transmembrane receptor involved in the immune and compliment systems. AD related mutations in TREM2 result in loss of function and are thought to mediate immunogenicity and inflammation that is enhanced in AD (Carmona et al., 2018). Myoclonus and seizures have been reported in a small cohort of carriers of the common TREM2 mutation R47H (Luis et al., 2014). Viral overexpression of TREM 2 was found to decrease pilocarpine induced seizures, while also reducing oxidative stress and increasing cell survival. TREM2s direct physiological role in AD has proven difficult to define, however, despite much investigation (Ulrich et al., 2017; Carmona et al., 2018). Likewise, its role in the induction of seizure pathology is yet to be detailed.

## Electroencephalographic Slowing and Hippocampal Hyperexcitability

The 3xTG mouse combines the APP Swedish mutation with the PSEN M146V mutation and the P301L mutation of Microtubule Associated Protein Tau (MAPT). The PSEN M146V mutation impairs γ-secretase cleavage of APP resulting in greater secretion of Aβ42 and increased Aβ42/Aβ40 ratio (Li et al., 2016). Individuals carrying this PSEN1 mutation have experienced onset of familial AD between 35 and 40 years with death in their early 50s (Clark et al., 1995; Riudavets et al., 2013). The MAPT P301L mutation is a missense point mutation in a conserved region of the gene encoding the microtubule protein tau. This mutation induces aggregation of tau protein in neurons and microglia, particularly in the hippocampus, neocortex, and substantia nigra leading to neuron loss and gliosis resulting in dementia and Parkinsonism, but notably this mutation is not specific to AD *per se* (Dumanchin et al., 1998; Hutton et al., 1998). When combined in the 3xTg mouse, these mutations lead to Aβ deposits by 6 months of age, age related tau aggregation after 6 months of age, and decreased LTP (Oddo et al., 2003). Gliosis has been detected at 7 months of age (Caruso et al., 2013) and impaired MWM and deficits in contextual fear conditioning emerges from 4 to 6 months (Billings et al., 2005).

Resting state functional magnetic imaging (re-fMRI) performed in 3xTg mice has revealed that, although tau immunoreactivity was present in 6–8 and 18–24 months old groups, the older cohort displays a higher degree of synchronous activity between multiple brain regions including hippocampus, auditory association cortex, temporal association cortex, and lateral entorhinal cortex. Thus, although development of tauopathy was not age-dependent in these mice, development of hypersynchronous activity in 3xTg is an age-dependent phenotype (Liu et al., 2018).

EEG slowing and hippocampal hyperexcitability are also AD-seizure relevant phenotypes in 3xTg mice. Juvenile 3xTg mice (postnatal day 30) present with increased hippocampal theta activity that is associated with increased tau phosphorylation. In these mice, this effect can be reversed by the potassium channel antagonist 4-aminopyradine (Mondragón-Rodríguez et al., 2018b). *In vivo* anesthetized recordings in older 4–6 and 17–18 months 3xTg mice comparing evoked responses in CA1 and DG of hippocampus following performant path stimulation revealed that short-term plasticity in response to brief high frequency stimulation is facilitated in both young and old 3xTg mice. Though responses to single or low frequency paired-pulses indicate that synapse integrity is nominal (Davis et al., 2014). This hippocampal hyperexcitability has been associated with pilocarpine-evoked (Yan et al., 2012), audiogenic (Kazim et al., 2017), and corneal kindled seizures (Vyver et al., 2020). This seizure susceptibility could be attenuated by treatment with an anti-human APP antibody or with an mGluR5 antagonist (Kazim et al., 2017). Though it remains to be determined if spontaneous seizure activity is present in 3xTg mice. Important to note are reports of inconsistent phenotypes related to behavioral and pathological changes in 3xTG mice thus again emphasizing the need for rigorous reporting of source and background strain (Onos et al., 2016).

Patch clamp recordings in hippocampal slices from 3 months old Tg2576 have revealed that overexpression of voltage gated sodium channels (NaV) leads to resting membrane depolarization and increased spike frequency. Interestingly, exposure of Tg2576 embryos to Aβ_1–42_ induced a selective upregulation of NaV 1.6 channels (Ciccone et al., 2019). In each case, it appears that neuronal hyperexcitability is present in Tg2576 mice at a very early age. This is coincident with an increase in theta power during REM (Kam et al., 2016), which is likely involved in the progressive cognitive impairments in Tg2576 mice.

Fu et al. (2019) demonstrated that epileptiform activity is detectable in J20 mice as early as 1 month of age and is associated with neuronal hyperexcitability. At 2 months of age these mice begin experiencing seizures that coincide with increases in hippocampal neurogenesis. This increased neurogenesis was not detected at 1 month of age. Decreased neurogenesis was then detected at 3 months of age compared to non-transgenic mice, which continued to decline over the lifespan of the mouse. These findings may indicate that neuronal hyperexcitability in the hippocampus drives early epileptiform activity, spurring an initial increase in neurogenesis in J20 mice. This neurogenesis feeds forward to produce seizures and deplete neural stem cells, leading to the advanced neurodegeneration in the J20 line. Treatment with levetiracetam was effective at blocking the increases in neurogenesis, rescue spatial discrimination deficits, and suppress seizures in J20 mice (Sanchez et al., 2012; Corbett et al., 2017). Notably, phase II clinical trials are currently underway to investigate the degree to which suppression of seizure related activity by levetiracetam can improve outcomes in AD (Levetiracetam for Alzheimer’s Disease-Associated Epileptiform Activity, 2020).

Seizure activity and the resulting cognitive deficits in J20 mice may be related to a deficient expression of calcium binding protein calbindin-D28k (You et al., 2017). Calbindin-D28k is a critical regulator of calcium signaling in the dentate gyrus (Brini et al., 2014) and expression of calbindin is inversely related to seizure activity in patients with temporal lobe epilepsy (Karádi et al., 2012). Calbindin expression was found to be decreased in both AD and non-AD dementia patients (McLachlan et al., 1987), is decreased in J20 mice (You et al., 2017), and knockout of calbindin-D28k results in deficient synaptic plasticity and spatial working memory impairment (Molinari et al., 1996). Transcription of the calbindin allele in the dentate gyrus is suppressed by promoter acetylation mediated by the activity driven expression of the immediate early gene ΔFosB. Seizure activity in J20 mice has been reported to be related to increased ΔFosB activity and reduced calbindin expression in the hippocampus (You et al., 2017). These markers coincide with decreased theta power and decreased time spent asleep, which predicted MWM deficits (Hazra et al., 2016).

A study in APP/PS1 mice compared induction of LTP in hippocampal slices of 1.5 and 8 months old mice following high frequency stimulation (HFS) of Schaffer collaterals. Small, but statistically non-significant differences in post HFS LTP were measured in 1.5 months old mice. At 8-months LTP was significantly diminished compared to younger mice. Smaller field excitatory post-synaptic potential (fEPSP) output in response to stimulation and increases in paired-pulse ratio at shorter inter-pulse intervals were also described. Treatment with the GABA agonist diazepam was sufficient to reduce paired-pulse ratios in 8 months old WT littermates, but did not produce a significant decrease in the APP/PS1 mice. Taken together, these studies indicate a loss of function in GABA signaling that produces dysfunction in hippocampal LTP (Oyelami et al., 2016), which may lead to hyperexcitability and age-dependent seizure and epileptiform activity in this strain (Reyes-Marin and Nuñez, 2017). In line with this observation, reductions in GABA currents, changes in GABA subunit expression, and a loss in sensitivity to GABA have been observed in post-mortem AD brains (Limon et al., 2012).

Multiple reports have characterized alterations in EEG power in 5xFAD mice. Siwek et al. (2015) reported an increase in hippocampal theta activity, but an overall decrease in mean theta frequency within that range. Both effects would be consistent with an EEG slowing phenotype. A separate report documented reductions in power across the delta-gamma range, but a substantial increase in power within the subdelta range (0.1–0.5 Hz) in 6 months old 5xFAD mice. This range of activity is not as frequently reported, but suggests a strong EEG slowing phenotype in 5xFAD (Schneider et al., 2014). Anesthetized field recordings in 8 months old 5xFAD have identified decreases in theta and gamma power elicited by brainstem stimulation (Stoiljkovic et al., 2016). Taken together, these data indicate neurophysiological deficiencies, especially within the theta frequency range, that are consistent with an EEG slowing phenotype in 5xFAD mice.

APOE4 mice were recently shown to have greater glucose metabolism in the entorhinal cortex measured by fMRI, increased activity of excitatory neurons in awake and freely moving mice, and longer duration extracellular field potentials with diminished LTP in hippocampal slices (Nuriel et al., 2017). These results provide strong evidence for an APOE4 related hyperexcitability phenotype in AD. Increased activity within the theta range has also been reported in APOE4 mice (Hunter et al., 2012). Though AD like progression associated with behavioral phenotypes are limited as deficits in spatial learning and memory are present in 3 months old mice (Rodriguez et al., 2013).

Rare gene variants of the disinterring and metalloproteinase domain-containing protein 10 (ADAM10) confer a low to moderate risk of AD. ADAM10 is an α-secretase that cleaves APP into non-toxic products preventing the production of Aβ fragments. Mutation or loss of function of ADAM10 will favor BACE cleavage and production of plaque forming Aβ fragments. Overexpression of ADAM10 in the hippocampus of C57BL/6J mice reduced markers of neuroinflammation (iNOS, COX-2, IL1-β, and TNF-α) and protected against pilocarpine-induced seizures (Zhu et al., 2018). On the other hand, ADAM10 knockout mice show increased activation of microglia, gliosis, and loss of spine morphology that coincided with a leftward shift of hippocampal EEG power in theta and gamma ranges, indicative of a hippocampal slowing phenotype (Prox et al., 2013). A direct relationship between ADAM10 mutation and seizure has yet to be described in humans but speaks to the utility of EEG findings in animal models that may be explored clinically.

Although not specific to AD, alpha-synuclein mutant mice used in the study of Dementia with Lewy Bodies (DLB) and other synucleinopathies may also be instructive to mechanisms related to seizure activity and hippocampal dysfunction. In DLB, inclusions of α-synuclein form when the presynaptic membrane is mislocalized and begins to aggregate (Morris et al., 2015). EEG slowing is a key biomarker shared by DLB and AD. In AD, EEG slowing is attributable to loss of acetylcholine neurons into hippocampus. This contributes to breakdowns in hippocampal plasticity and dysregulation of recurrent excitatory/inhibitory circuitry between the cortex. Intermittent seizure activity with myoclonic jerks and EEG featuring prominent ictal spikes were evident in mice overexpressing human α-synuclein. These events coincided with a leftward shift in baseline EEG power reflecting a greater proportion of low frequency activity (Morris et al., 2015). When overexpression of a mutant transgene of α-synuclein (A53S) is combined in a mouse lacking endogenous tau, EEG-slowing induced by overexpression of α-synuclein is markedly attenuated, suggesting a clear link between these two proteins in seizure pathology witnessed in DLB (Peters et al., 2020).

The AD-relevant neural activity has also been identified in some rat models as well. F344-AD rats express two transgenes: Swedish (APP KN670/671NL) and human presenilin PS1ΔE9. Urethane anesthetized and freely moving F344-AD rats exhibit diminished activity within alpha frequencies. Though, diminished activity was also observed in the theta frequency range, opposite of what is typically seen for EEG slowing. In both WT and F344-AD anesthetized rats, stimulation of the tegmental nucleus pontis oralis at 6 months of age strongly entrains a theta oscillation in the hippocampus. At 12 months, however, this evoked theta oscillation is diminished (Stoiljkovic et al., 2019). Treatment with the acetylcholinesterase inhibitor donepezil was able to restore evoked and freely-moving theta oscillations in F344-AD rats, while augmenting this activity in WT counterparts (Stoiljkovic et al., 2018). In patients with mild dementia, however, long-term treatment with donepezil reduces absolute-theta activity. So it is likely the beneficial effects of donepezil in patients reflects a long-term response to treatment (Kogan et al., 2001).

While the majority of EEG phenotypes in AD animal models have been reported in rodent species including in this present review, despite the utility of EEG in rodents for translational studies, it should not be dismissed that neurophysiological characteristics of rodents are largely different from those of humans. Thus, most pharmaceutical interventions that have been developed in rodents and reported as efficacious for improving cognitive impairment did not show any significant cognitive improvement in clinical trials (Silverman et al., 2020). From this perspective, the use of NHPs as animal models for AD have been of growing interest because of the maturity and anatomical similarity of the primate brain to those of humans (Kaiser and Feng, 2015; Van Essen et al., 2019). It has been demonstrated that aggregation of Aβ plaques which is similar in sequence to humans were naturally found in aged animals accompanied by declining cognitive function (Geula et al., 2002; Sani et al., 2003; Oikawa et al., 2010). In NHPs, some AD models have already been published including rhesus monkeys, cynomolgus monkeys, vervet monkeys, and marmosets (Geula et al., 1998, 2002; Lemere et al., 2004; Forny-Germano et al., 2014; Yang et al., 2014; Melamed et al., 2017; Seita et al., 2020). Details of each model were described elsewhere but it is noteworthy that recent technological advances have made it possible to generate genetically engineered animal models in NHPs (Sasaki et al., 2009; De Felice and Munoz, 2016; Li et al., 2019; Seita et al., 2020). There are still a number of limitations when utilizing NHPs as AD models such as their relatively long lifespans compared to rodents as well as ethical and technical issues. While the utility for conducting EEG across NHP species has been demonstrated (Muggleton et al., 2005; Authier et al., 2014; Cheng et al., 2015; Nakazawa et al., 2015; Ishikawa et al., 2017; Uslaner et al., 2018), to the best our knowledge, only one comprehensive study investigating epileptiform activity in an AD relevant NHP model has been reported; reporting intriguing results of EEG signatures with corresponding pathology and cognitive decline in mouse lemurs (Gary et al., 2019). Further studies with NHPs could reveal the roles of epileptiform activity of neurons in AD and accelerate the understanding of the etiology and progressive mechanism of this disease, as well as provide primate specific translational outcomes for AD.

## Theta-Gamma Interactions

TGC is impaired in App*^NL–F^* mice. Local field potentials were recorded in 5 months old homozygous male and female App*^NL–F^* and five non-litter male and female C57BL/6 mice using tetrodes implanted and advanced through the entorhinal cortex. TGC was measured as the phase at the time of gamma oscillation maxima. Prominent gamma oscillations were characterized during theta states in both strains. Further the power of gamma oscillations did not differ between strains. However, though the number of gamma oscillations were not significantly affected in App*^NL–F^* mice the temporal structure of gamma oscillations was impaired. Specifically, the peak of gamma oscillations observed in the falling phase to trough of theta was reduced in magnitude compared to C57BL/6. So, while epileptiform activity may be absent in App*^NL–F^* mice, they are not spared in terms of EEG abnormalities (Nakazono et al., 2017).

Alterations in spike-gamma coupling have been observed in App*^NL–G–F^* mice. Spike-gamma coupling refers to the generation of gamma oscillations generated by rhythmic synchronous activity of fast spiking interneurons. A loss of spike-gamma coupling could lead to impairments in theta-gamma coupling as temporal associations breakdown across oscillatory frequencies. Patch clamp recordings of fast spiking interneurons and pyramidal cells in 1, 2, 4, and 6 months old. App*^NL–G–F^* mice revealed impairment of spike-gamma coherence as early as 2 months of age, preceding amyloid plaque formation. Gamma oscillations also become degraded with age in App*^NL–G–F^* mice, which is correlated to Aβ_1–42_ concentration in the brain (Arroyo-García et al., 2021). This result suggests that the breakdown of gamma oscillatory activity and coupling to fast-spiking interneurons in App*^NL–G–F^* mice may contribute to breakdown of theta-gamma interactions, though this remains to be demonstrated.

J20 mice also exhibit breakdowns in cross frequency coupling. In an *in vitro* prep combining patch and field recordings with optogenetics in hippocampal tissue from 30 to 39 day old mice, a number of circuit-level abnormalities were characterized. First, theta oscillations displayed less rhythmicity compared to non-transgenic mice. TG mice also exhibited reduced theta-gamma CFC. Therefore it is clear that these circuit level disruptions are detectable in very young J20 mice, preceding any signs of Aβ accumulation (Mondragón-Rodríguez et al., 2018a). J20 mice expressing re-recombinase (PVJ20) to enable optogenetic interventions exhibit decreased slow gamma (30–60 Hz) coupling to theta. Gamma oscillation power was also reduced in the PVJ20 mice compared to PVJ20- littermates. Optogenetically mediated 40 Hz stimulation was effective in increasing slow gamma phase-amplitude coupling to theta. However, 80 Hz stimulation did not increase coupling, possibly because fast-gamma coupling was not impaired. Further, 40 Hz stimulation rescued a deficit in discrimination in a novel object place recognition paradigm (Etter et al., 2019). These results suggest that optogenetic stimulation in the slow gamma frequency range is capable of restoring gamma oscillations and improving deficient memory recall.

3xTg mice also exhibit deficient coordination between theta, slow gamma, and place cells in the hippocampus at 8–9 months of age. Mice were trained to run unidirectionally around a circular track to receive a reward that was always placed in the same location. Tetrodes aimed at the CA1 region of hippocampus recorded single units and continuous sampled local field potentials. Theta phase coupled increases in slow gamma power were diminished in 3xTG mice compared to WTs, suggesting that hippocampal slow gamma modulation is impaired in the 3xTg mouse (Mably et al., 2017). Though it is noteworthy that amyloid deposition was not detected in the 8–9 month 3xTg mice in this study, which contradicts earlier findings that amyloid is detectable in this strain as early as 4 months (Oddo et al., 2003).

Transgenic rats also exhibit impaired CFC. Anesthetized *in vivo* recordings in the hippocampus and mPFC of 9-month old F344-AD rats assessed phase-amplitude coupling, specifically the ability of low frequency theta oscillations to modulate high frequency gamma oscillations. This analysis showed a decrease in phase-amplitude CFC in both regions (Bazzigaluppi et al., 2018). These results suggest poor temporal coordination within these networks in this strain.

## Discussion

Subclinical seizure and epileptiform activity are potentially very powerful pre-diagnostic biomarkers of prodromal dementia and LOAD (Vossel et al., 2013, 2016, 2017; Horváth et al., 2016; Palop and Mucke, 2016; Vöglein et al., 2020). Four distinct phenotypes have repeatedly emerged in the course of this and other reviews, which could potentially be assessed both in patient populations and mouse models using relatively inexpensive and accessible tools. These emergent phenotypes are (1) ectopic cortical spiking activity, especially during NREM sleep, absent overt physical symptoms of acute seizure, (2) hippocampal slowing evidenced by leftward shifts in the power distribution of EEG oscillatory activity at baseline, (3) hyperexcitability of hippocampal neurons, particularly in response to cortical inputs, and (4) theta-gamma interactions. In patient populations, this activity is not only highly predictive of disease course (Vöglein et al., 2020), but may precede the earliest cognitive symptoms in LOAD by >5 years (Vossel et al., 2013, 2017). Increased preventative screening could push this boundary even earlier and allow for aggressive intervention and cognitive therapy while also aiding family and caretakers in the preparation and planning of disease course. Absent any further advancement, these factors alone would dramatically improve quality of life of patients, loved ones, and caretakers, as well as the general population as a whole. With continued definition, methodological advancement, and standardization, regular EEG assessment of these phenotypes in conjunction with genetic screening for risk alleles could become a standard of preventative care in individuals over the age of 40.

Aside from clinical value, these EEG biomarkers have high potential to provide insights into the etiology and progression of AD in ways that go beyond amyloid and tau hypotheses that have failed to produce tenable, disease-modifying treatments for AD. In rodents, EEG and EMG can be recorded by wireless telemetric transmitters using skull surface electrodes that can be implanted with a relatively simple surgical preparation (Vogel et al., 2002; Kramer and Kinter, 2003; Lundt et al., 2015). This can be combined with depth electrodes throughout related circuitry (Gureviciene et al., 2019). Analysis of these data sets can also be largely automated. This preparation allows for minimally invasive, continual assessment of epileptiform activity. There is also value added in sleep analysis in the same animal. As has been noted in this and earlier reviews (Born, 2015; Palop and Mucke, 2016; Vossel et al., 2017; Horvath, 2018), nearly equivalent measures of human seizure and epileptiform activity are detectible in model mice for AD. These measures are sensitive to acetylcholinesterase inhibitors (Reyes-Marin and Nuñez, 2017; Stoiljkovic et al., 2018) and anti-seizure (Sanchez et al., 2012) medications utilized in the treatment of AD. Taken together, this is evidence of high construct, face, and predictive validity of these biomarkers. Understanding of the origins and physiology of epileptiform activity in model animals has the potential to broaden horizons for novel treatments in AD.

There is plenty of room for improvement in this arena. First, there is no animal model that fully recapitulates all aspects of AD; an important caveat that has been discussed extensively elsewhere (see Jankowsky and Zheng, 2017; Oblak et al., 2020; Mckean et al., 2021; Vitek et al., 2021). Although these animal models continue to be powerful tools for forming our current understanding of AD and for translational studies evaluating potential therapeutic interventions, most of the rodent models evaluated for EEG phenotypes to date are primarily transgenic mouse models based on familial, early onset mutations. The models present with aggressive amyloid or tau overexpression that manifests at an early age (e.g., 6 months). Presentation of disease in 6–12 months aged animals is merely equivalent to that of a 20–50 years old human and therefore does not capture physiological changes related to aging which are critical for the study of neurodegenerative diseases including AD. While minimizing aging costs for research colonies of aging animal models may be a benefit in the short term to researchers, incorporating the biological construct of aging is important for improving preclinical to clinical translation and represents a major pitfall which has plagued the AD animal model field. Relatedly, the lack of applying standardized nomenclature of these mouse lines further complicates an already dense literature. Mouse models are frequently referred to with shorthanded nomenclature which do not necessarily include the specific mutation or background strain information (Sukoff Rizzo et al., 2020), Examples include whether the strain is overexpressing a transgene, if sequence of alleles are humanized, and if the mice are congenic or a hybrid cross of multiple strains. Importantly, the background strains themselves have divergent EEG signatures, which may contribute to the phenotype of the mutation and are often under-reported (Copping et al., 2019). These pitfalls may lead to misidentification of similarities between different strains or under appreciation of overlap where it does exist. Ensuring that standardized nomenclature is applied when reporting data from mouse models continues to be critical and requires much consideration by researchers.

Second, understanding of the genetic mechanisms and environmental interactions contributing to LOAD are still poorly understood. Third, understanding of the timecourse of progressive degeneration and normal aging related changes in EEG in animal models has been limited. Finally, putative disease modifying drugs developed using these mice have all thus far failed to translate (Silverman et al., 2020). If the field is to realize the power of EEG as an important translational biomarker there is an onus on researchers to begin incorporating the key risk factors for AD, of aging and LOAD risk genes (Onos et al., 2016; Oblak et al., 2020).

Ideally, these EEG measures will begin to inform the onset of pathological changes in AD and predict cognitive decline, which has thus far eluded researchers to date. To date, EEG as an early biomarker has not yet fully realized its potential with respect to cognitive changes. Importantly, detection of spontaneous sub-clinical seizures may be difficult in a clinical setting where it may not be possible to record for extensive periods of time, which could limit its potential. Optimally, future investigations can begin incorporating EEG in animals performing cognitive tasks with discrete stimulus presentation and high temporal fidelity with recordings, such as the touchscreen batteries that are rapidly becoming increasingly commonplace in behavioral neuroscience (Horner et al., 2013; Marquardt et al., 2017). These active tasks can provide finer-grain analysis of pathophysiology in AD, potentially across a shorter period. Indeed, touchscreen tasks have already been validated in App^*NL*–*G*–*F/NL*–*G*–*F*^ mice (Saifullah et al., 2020) and methods to pair these tasks with EEG have already been detailed (Marquardt et al., 2017). These methods also have the potential to improve translation between rodents and humans by putatively equalizing stimulus detection and response strategies across species to eliminate potential confounds.

The specificity of EEG signatures as biomarkers continues to remain under speculation across the field and requires more research. Measures of electroencephalographic hyperexcitability have been reported in several CNS disorders including in multiple sclerosis (Zipser et al., 2018), autism spectrum disorders (Boutros et al., 2015), and Parkinson’s disease (Yu et al., 2007), among others. Indeed more work is required in order to determine the distinct contributions of AD related pathology in the pathogenesis of these EEG biomarkers and specific AD related signatures. In particular, it will be very important that future endeavors understand time course of these markers. In animal models, many have been characterized very early on, even before significant amyloid deposition. Therefore, prospective, longitudinal studies would be key in parsing the specific contributions of these EEG biomarkers to the pathogenesis of age-related cognitive decline in AD.

Overall, pre-clinical AD research is in need of new approaches. Decades of research focused on behavioral testing in FAD mutant mouse models have not appreciably moved the needle on our ability to prevent degeneration or extend lifespan in patients. EEG assessment of epileptiform activity in diverse model systems including NHPs and new models genetically engineered with mutations relevant to LOAD risk has the potential to push this horizon in new ways and ultimately enable accelerating the pace of bringing effective therapeutics to patients.

## Author Contributions

All authors listed have made a substantial, direct, and intellectual contribution to the work, and approved it for publication.

## Conflict of Interest

The authors declare that the research was conducted in the absence of any commercial or financial relationships that could be construed as a potential conflict of interest.

## Publisher’s Note

All claims expressed in this article are solely those of the authors and do not necessarily represent those of their affiliated organizations, or those of the publisher, the editors and the reviewers. Any product that may be evaluated in this article, or claim that may be made by its manufacturer, is not guaranteed or endorsed by the publisher.
